# Job Crafting and Performance in Firefighters: The Role of Work Meaning and Work Engagement

**DOI:** 10.3389/fpsyg.2020.00894

**Published:** 2020-05-20

**Authors:** Cristina-Ioana Dan, Andra Cǎtǎlina Roşca, Alexandru Mateizer

**Affiliations:** ^1^Centre for Psychosociology, Ministry of Internal Affairs, Bucharest, Romania; ^2^Department of Sociology-Psychology, Faculty of Political Sciences, National University of Political Sciences and Public Administration, Bucharest, Romania; ^3^Faculty of Psychology and Educational Sciences, University of Bucharest, Bucharest, Romania

**Keywords:** JD-R, job crafting, work engagement, work meaning, performance, firefighters, first responders

## Abstract

The Job Demands-Resources (JD-R) model was often utilized as an explanatory framework when investigating the strain process among first responders in general and firefighters in particular. Yet, little is known about the motivational processes whithin firefighters. The aim of this study is to expand the knowledge regarding the motivational process of firefighters by investigating job crafting and introducing work meaning within the motivational framework of the JD-R model, in relation to job performance. A cross-sectional survey design was used to collect data from one sample consisting of Romanian firefighters (*n* = 1,151). Structural equation modeling indicated the existence of both a direct and an indirect effect between job crafting and job performance through work meaning and work engagement. Our findings suggest that firefighters actively engage in job crafting behaviors and also that work meaning can be an outcome of job crafting. Results also encourage further research related to the way work meaning impacts job performance, through its link with work engagement. This study raises attention on how Fire departments may be able to create a climate that emphasizes meaningfulness and engagement, together with opportunities toward job redesign and a focus process based on efficiency gain.

## Introduction

Firefighting personnel have to deal on a daily basis with major strains, including life-threatening missions, exposure to emotional and physical trauma, adverse weather conditions (heat, cold, and noise) and hazardous materials. In this context, alongside efforts in managing specific strains, supporting the motivational drive for the firefighting personnel becomes imperative in sustaining a positive job performance outcome. This article adds to the literature by investigating how job crafting behaviors in firefighters can contribute to work engagement, personal work meaning and performance.

It is agreed that, in their everyday work, firefighters encounter tasks that can be life-threatening, thus having a high impairment potential. For instance, one issue was identified as being the moral suffering and compassion fatigue as secondary traumatic stress in first responders (Papazoglou and Chopko, 2017; Papazoglou and Tuttle, 2018). However, we are dealing with pre-selected employees, due to screening-out psychological strategies and consequently we expect that the resilience factors in firefighters are higher than in the general population (Galatzer-Levy et al., 2013). Still, keeping in mind the strains of their work and the body of knowledge related to this issue, we can also focus on ways in which to better sustain the motivational process of firefighters.

This focus might suggest practical approaches for the psychological training and support of firefighters and it could also inform on how the organizational climate could adapt in order to become more responsive to their needs, in ways that could make them ready to invest their resources in work and maintain an adequate level of engagement.

Due to their specific dangerous missions, literature has focused mainly on the strains that firefighters face rather than on the positive aspects underlying their specific tasks. The Job Demands-Resources (JD-R) model (Demerouti et al., 2001) was often used as an explanatory theory when investigating the strain process among firefighters (Ângelo and Chambel, 2014; Airila, 2015). The assumptions of the JD-R model are focused on two different processes, health-impairment and motivational, which employ work characteristics as factors that have either a positive or a negative impact on employee well-being (Schaufeli and Bakker, 2004). Few studies approached the positive dimensions of firefighter’s work, and those that did have focused mainly on engagement (Ângelo and Chambel, 2014) and post-traumatic growth (Armstrong et al., 2014).

Firefighters are organized as a military structure and are thus subjected to military rules and rigid regulations and orders. Thus, it could be easy to assume that their work would be not susceptible to job crafting in its classical top-down approach (e.g., simplification, standardization, or enrichment) (Oldham and Fried, 2016). This is probably one explanation of why job crafting was not approached, until now, in studies involving firefighters. Also, even if we consider work meaning to be particularly important in firefighters, due to the fact that their work has intrinsic positive significance for the personnel and society as a whole, this concept and its role in the motivational process of firefighters has not yet been investigated.

Still, understanding the deeper dynamics and implications of job crafting could lead us to an approach that takes into consideration the meaning that employees can create by themselves, the interactions that they seek and the relational boundaries they create. Within the military teams of the firefighter’s units, tasks are clearly specified, procedures and regulations are strict. But social bonding is created alongside social regulations imposed by military rules (such as the communication chain, salutation, and reporting to a superior). The team becomes their second home, it is the place where firefighters feel secure to share experiences, tactics, and emotions. In this interactional process, they maintain meaning, but they also create meaning and shape their daily work.

These aspects are leading us to considering job crafting as related to work meaning in the process of sustaining engagement and consequently job performance in firefighters. In this study we aim to expand the knowledge regarding the motivational process of firefighters by employing job crafting and introducing the concept of work meaning within the motivational framework of the JD-R model (Demerouti et al., 2001). We expect participants that engage in job crafting behaviors to have a greater sense of work meaning, to be more engaged in their work and to perform well.

This article may contribute to the literature in three ways. First, by focusing on the motivational dimension in firefighters. Second, we investigate the measure in which job crafting behaviors exist within the military structure of firefighters. Can firefighters actively engage in shaping their work? And lastly, by seeking to expand the JD-R model’s motivational process with the introduction of work meaning as a contributing factor to work engagement and job performance.

### Job Crafting

Job crafting consists in the physical and cognitive changes individuals make in the tasks they face, in the relational boundaries of their work or in the way they think about their job, in an attempt to adapt their work environment (Xanthopoulou et al., 2007). This process is possible since job boundaries, meaning of work, and work identities are not fully determined by formal job requirements (Wrzesniewski and Dutton, 2001; Berg et al., 2013). A job is comprised of tasks and relations assigned to one individual in an organization, under one job title (Ilgen and Hollenbeck, 1991). Interactions with others at the workplace help employees understand the boundaries of their job, and also help shape impressions and relationships with others within the work environment (Wrzesniewski and Dutton, 2001). Recent studies have suggested the existence of other dimensions of job crafting as well. It has been found that employees actively searched for challenges in their work (Bakker et al., 2012) or engaged in behaviors that pertain to their skill development (Lyons, 2008).

In this article we employ the framework developed by Tims et al. (2012) where job crafting is defined as the changes related to job demands and resources that employees may initiate. This conceptualization is embedded in the JD-R model (Demerouti et al., 2001) which places all job characteristics into two separate categories, demands and resources. Job demands are job aspects (physical, social, and organizational) requiring sustained physical and/or psychological effort, associated with physiological and/or psychological costs (Demerouti et al., 2001). Job resources, such as autonomy, opportunities for growth and performance feedback, are physical, social, psychological, and/or organizational aspects of the job important in the achievement of organizational goals, able to reduce job demands and to stimulate personal growth and development (Demerouti et al., 2001). The empirical evidence for JD-R model is very rich, constantly demonstraiting the relations between its variables.

Within this context, according to Tims et al. (2012) job crafting consists of four different types of behaviors: increasing structural job resources, increasing social job resources, increasing challenging job demands and decreasing hindering job demands. In this article we focused on the first three dimensions as we were aiming to investigate the rellationships between job crafting, work meaning, engagement, and job performance. Also, previous research has shown that decreasing hindering job demands is ineffective, as it is negatively associated with organizational outcomes such as job performance (Tims et al., 2012; Gordon et al., 2015). Given the dangerous nature of firefighters work and their strong reliance on team effort and sense of group identity we expect individuals in our sample to be engaged in interactions that encourage collaboration and fedback (social resources), to actively participate in activities that ensure their skill variety and development (e.g., trainings and structural resources) and to search for challenges in order to stay engaged and performant in their work.

### Job Crafting and Work Meaning

Different definitions of work meaning have been forwarded by scholars, and a lack of consensus still surrounds the inner mechanisms of this concept. The issue of work meaning is regarded by some as two-fold, the term “meaning” comprising both an objective (what work signifies) and subjective (the personal relevance of what work signifies, e.g., meaningfulness) dimensions. This differentiation is emphasized by some authors (see Pratt and Ashforth, 2003) while others (Wrzesniewski et al., 2013) mention the dynamical relationship between these two aspects. Following Rosso et al. (2010) we regard work meaning not only in its factual dimension but also as representing work that is significant and meaningful for the individual. For the purpose of this article, we will use Steger et al. (2012) definition of work meaning as the subjective experience that one’s own work has positive significance, facilitates personal growth, and contributes to the greater good. We will use the terms “meaning” and “meaningfulness” interchangeably as our theoretical stance proposes that meaningfulness of work is the subjective correspondent of meaning of work.

Meaningful work arises when people have a clear sense of self, an accurate understanding of the nature and expectations of their work environment, and the ability to transact with their organizations in order to accomplish their work objectives (Steger and Dik, 2009). Vuori et al. (2012) indicate three means by which employees extract meaning at work: emphasizing positive aspects of work, developing competencies in order to enhance performance or a positive reaction from co-workers and influencing work contents. According to Rosso et al. (2010) the sources of meaning at work are the self, others, the work context and spiritual life. Following the idea of work context, job characteristics are important in shaping employees’ perception of the meaningfulness of their work (Wrzesniewski and Dutton, 2001). Furthermore, the positive significance of tasks characterized by a sense of purpose and positive impact was found to be linked to more sense of meaning (Grant, 2008). Meaningfulness of one’s work may provide individuals with a sense of fulfillment and also drive toward dedicating time and effort on work-related activities (Wrzesniewski et al., 1997).

Recent work in this domain has put forward the idea that work meaning is not only derived from job characteristics but also from the individual’s proactive efforts to redesign his tasks and relational dynamics within the work environment (Leana et al., 2009; Berg et al., 2010; Rosso et al., 2010). This perspective is one that extends the assumptions of traditional job design research and was further attested in a longitudinal study by Tims et al. (2015). The results of their analysis indicated that work meaning can be an outcome of job crafting. Furthermore, according to Bakker et al. (2012) an organizational climate that supports and challenges the employee and is, at the same time, responsive to their needs, increases the likelihood that employees will become more willing to invest time and energy at work and to be more engaged in their work. We would also argue that such an environment activates the individual’s sense of purpose, personal meaning, and agency.

For firefighters, we consider work meaning particularly important, due to the fact that their work has intrinsic positive value and significance for employees and society as a whole. Work is the place where firefighters create and maintain meaning in their work through group interaction and group identity (Katz and Kahn, 1978; Weick, 1995). On the basis of this literature review we argue that individuals who craft their jobs will have a greater sense of work meaning. Therefore, we formulated our first hypothesis.

Hypothesis 1. Job crafting is positively related to work meaning.

### Job Crafting and Work Engagement

The concept of work engagement is a measure of the way employees perceive and experience their work. According to Schaufeli et al. (2002), work engagement refers to a state of mind related to work that can be described by three dimensions, vigor, dedication, and absorption. In other words, work is viewed as stimulating, as significant and worthwhile and as captivating and immersive. This conceptualization of work engagement is adopted within the JD-R model (Demerouti et al., 2001) and we will use it for the purpose of this article since it is focused on the actual activities that one deals with at work.

This concept relies strongly on a motivational perspective that is highly dependent on the outcomes of a dynamic process of interaction between the work environment, job characteristics and personal work aims and resources. Literature shows that job resources, such as feedback, social support and skill variety, are assumed to play both extrinsic and intrinsic motivational roles, given that they are pivotal in achieving job related goals and also in fostering employees’ learning and development (Bakker et al., 2012). Research has found that under specific conditions employees’ engagement can be at an optimum level. Hakanen et al. (2005) showed that appropriate and sufficient job resources coupled with high challenges are associated with greater levels of engagement. Based on these considerations we can conclude that the changes one makes to the work environment (e.g., job crafting), that can be translated into more resources and challenges, can lead to an increase in work engagement. In their article, Bakker et al. (2012) have shown that this relationship holds among employees from organizations in Netherlands. Also, in another study conducted by Tims et al. (2013b) in a large Occupational Health Services company in Netherlands (*N* = 562), results showed that job crafting can be an important strategy for increasing work engagement and job performance, at both individual and team levels.

The relationship between job crafting and work engagement is most certainly a dynamic one (Bakker and Bal, 2010). Positive affect and involvement associated with dimensions of work engagement may promote an active approach in the work place. According to Bakker et al. (2012), engaged employees are more likely to behave in a proactive manner because they have an increased ability to think innovatively and to see possibilities for problem solving.

In this article we pursue the effect that job crafting has on work engagement and we argue that individuals who adopt such behaviors will show increased engagement in their work. Based on these considerations we expect to find such an effect within firefighters. By increasing their structural and social resources and by endorsing new challenges within their domain of activity firefighters may sustain an ongoing process of regulating their levels of work engagement. Thus, we formulated our next hypothesis.

Hypothesis 2. Job crafting is positively related to work engagement.

### Work Meaning and Work Engagement

Individuals who positively value their work and find sources of personal meaning through work are, as a consequence, more interested and dedicated to their domain of activity (Britt et al., 2007). Integration of work as an identity related factor is accompanied by sense of purpose and opportunity to attest core self-values (Sheldon and Elliot, 1999). These considerations regarding work meaning can describe a mechanism through which the motivation for work engagement and consequent efforts at betterment are maintained.

Previous research has linked work meaning to a series of positive organizational outcomes such as higher job performance and productivity (Bunderson and Thompson, 2009; Rosso et al., 2010) or greater organizational commitment and work engagement (Duffy et al., 2013a, b; Geldenhuys et al., 2014). For example, a study by Beukes and Botha (2013), conducted on 199 nurses from private hospitals in South Africa, revealed that participants who found their work highly meaningful were also more likely to be engaged in their work and more committed to the organization. Also, Van Zyl et al. (2010) found that, for a convenience sample of South African psychologists (*N* = 106), meaningfulness of work, either experienced as a calling or as a consequence of job crafting and work-role fit, was strongly related to work engagement.

Considering the job characteristics of firefighters, where danger, risk and dedication are crucial aspects in fighting for a greater good, we expect work meaning to play an important role in their work engagement level. By focusing on their personal meaning at work, on work as a mean of creating meaning, on the desire to contribute to a greater good (Steger et al., 2012) and also on the personal significance of their group identity, we expect firefighters to be more engaged in their work. Based on these considerations we formulated our next hypothesis.

Hypothesis 3. meaning is positively related to work engagement.

So far, the theoretical and empirical arguments exposed in this article suggest the possibility of a partial mediation effect from job crafting to work engagement via work meaning. By engaging in job crafting behaviors firefighter personnel may obtain a greater sense of meaningfulness and maintain a necessary level of engagement that is critical in the exercise of their work. This leads to our next hypothesis.

Hypothesis 4. Work meaning partially mediates the relationship between job crafting and work engagement.

### Job Performance and Contributing Factors

In this article we employed job performance as the two-dimensional concept used within the JD-R model. According to Williams and Anderson (1991), in-role performance relates to activities that are in line with organizational goals and functioning and can be considered as part of the job description. On the other hand, extra-role performance is intrinsically motivated and supports a healthy (social) work climate.

Several studies have shown a positive relationship between job crating and job performance. This link may be explained by the fact that job crafting could be used to acquire resources that can be invested in job performance aspects and also, that it can lead to the development of abilities in performing more complex tasks by increasing challenges (Tims et al., 2013a). For example, Gordon et al. (2015) found that job crafting positively influenced performance in groups of health care professionals from the United States and Netherlands. Results showed that changes in work initiated by individuals through adjusting the job in order to fit personal work preference and seeking resources and challenges are beneficial to both organization and employees. In another study, Lyons (2008) found a positive association between job crafting endeavors and increases in performance in 107 outside salespersons. Furthermore, findings in a meta-analysis by Rudolph et al. (2017) revealed that increasing structural job resources was the most important dimension of job crafting that contributes to performance outcomes. Given these aspects we expect that, in our sample, job crafting will have a direct effect on performance.

Hypothesis 5. Job crafting is positively related to job performance.

One of the most convincing arguments for the reason why engaged workers show better performance outcomes than less engaged workers (Demerouti and Cropanzano, 2010) is that engaged individuals experience more positive emotions, including happiness, joy, and enthusiasm (Bakker et al., 2012). In turn, these positive emotions can lead to more flexible thought processes and actions (Fredrickson, 2001) thus increasing their chances of performance. The theorized connection between positive emotions and job performance outcomes is also partly explained by the consequent activation and expansion of personal resources (Fredrickson, 2001), such as physical, social, or psychological resources. These personal resources can be employed to better manage and cope with job demands and to attain better performance results (Bakker and Xanthopoulou, 2009; Luthans et al., 2010). Several studies have attested the positive relationship between work engagement and job performance. For example, Bakker et al. (2012) have found that, for a sample of general employees in organizations from Netherlands (*N* = 190), this relationship holds even when job performance was measured by using colleague-ratings instead of self-reports. Also, in their study Tims et al. (2013b) revealed that work engagement, and more importantly the vigor dimension, was related to job performance, concluding that the energetic aspect of engagement is most likely to facilitate goal-directed behavior.

Work engagement strengthens organizational commitment (Bakker and Bal, 2010; Bakker et al., 2014) and performance in business units (Harter et al., 2002), being positively related to objective task performance (Yongxing et al., 2017). In conclusion, work engagement is adding energy and persistence to employees, leading them to a better overall job performance. In our study we expect to find a direct relationship between work engagement and performance.

Hypothesis 6. Work engagement is positively related to job performance.

Our next hypothesis derives from hypotheses 2, 3, 5, and 6. We expect job crafting to also indirectly relate to job performance through its relationship with work engagement. In sustaining this idea, the meta-analysis of Oprea et al. (2019), showed that the effects of job crafting on performance were explained by increases in work engagement. In other words, we expect a partial mediation effect between job crafting and job performance. Empirical support for this claim can be found in previous studies (Bakker et al., 2012; Tims et al., 2013b) that showed the plausibility of this effect. For example, Tims et al. (2013b) found evidence for the existence of an indirect effect from job crafting to performance by way of work engagement, more precisely the vigor dimension. We anticipate a similar effect, therefore, our next hypothesis is as follows.

Hypothesis 7. Work engagement partially mediates the relationship between job crafting and job performance.

We also expect that work meaning exerts an effect on job performance through its positive association with work engagement. Driven by their personal meaning at work, by work as a mean of creating meaning, by the desire to contribute to a greater good (Steger et al., 2012) and also by the personal significance of their group identity, we expect firefighters to be more engaged in their work and to be more able to sustain performance.

Hypothesis 8. Work engagement mediates the relationship between work meaning and job performance.

**FIGURE 1:**
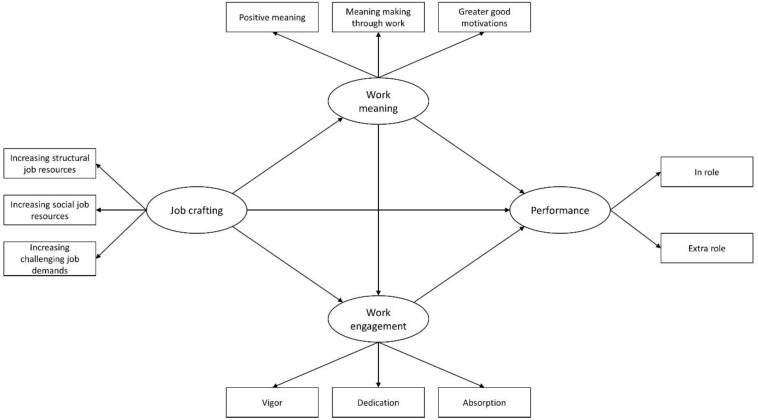
Hypothesized relationships of the proposed research model. All predicted relationships are positive.

In brief, the present article is focusing on factors that are contributing to the motivational process of the JD-R model in firefighters. We found good theoretical and empirical support for the role of work meaning in organizational outcomes and based on the JD-R model we will employ work meaning as a factor that positively impacts job performance. This approach extends existing literature on the JD-R model and firefighters first by focusing on the motivational process of JD-R model in firefighters, secondly by investigating job crafting within a military structure, and lastly, by examining the role of work meaning in attaining performance. Our assumptions were translated in the proposed research model, illustrated in Figure 1.

## Materials and Methods

### Participants and Procedure

We conducted our research based on a sample of 1,151 firefighters from 27 Romanian fire departments which were recruited by unit psychologists and invited to fill out a paper-and-pencil questionnaire, on a voluntary basis. The selection process was aimed at covering all types of interventions assigned to Romanian firefighters’ units. Unit psychologists conducted the questionnaires’ application process (without the researchers’ direct participation during administration), based on the informed consent of the participants. They emphasized the confidentiality and anonymity aspects of participation and also motivated respondents for sincere and open responses. In our sample, 99.2% of respondents were men and 0.8% were women. The mean age of the participants was 39.03 years (*SD* = 6.9; range: 20–58 years). A total of 71.3% of the participants had a non-leadership position and 28.7% had a leadership position. A rate of 61.5% held a high school diploma and 38.5% had a university degree. The majority of participants were married or in a relationship (81.6%) while 17.7% were single or divorced. Regarding the level of seniority, 68.8% of the participants were in service for 10 to 15 years, 11.2% were in service for more than 20 years, 3.3% were in service for 5 to 10 years, and 7.9% from 0 to 5 years.

### Measures

*Work meaning* (WM) was measured with The Work and Meaning Inventory (WAMI) (Steger et al., 2012), a self-report instrument that includes three dimensions: positive meaning (e.g., “I have found a meaningful career”), consisting of four items, meaning making through work (e.g., “I view my work as contributing to my personal growth”), and greater good motivations (e.g., “I know my work makes a positive difference in the world.”), each consisting of three items. For the Greater good motivations subscale one item was reversed (“My work really makes no difference to the world”). The ten items of WAMI are rated on a 5-point Likert scale, ranging from, from 1 (absolutely untrue) to 5 (absolutely true). The subscale scores are obtained by adding the corresponding item scores. The authors suggest that the subscales are highly intercorrelated (0.65–0.78) (Steger et al., 2012). For our sample, we used the Romanian version of the WAMI, translated from English, through a retroversion process. To our knowledge, the questionnaire was not used before on Romanian respondents. The reliability analysis we performed on our sample revealed a Cronbach’s alpha coefficient of 0.885.

*Work engagement* (WE) was operationalized with the Utrecht Work Engagement Scale – Short Version (UWES-9) (Schaufeli et al., 2006), a self-report instrument that includes three dimensions each of which consists of three items: vigor (e.g., “At my work, I feel bursting with energy”), dedication (e.g., “I am enthusiastic about my job”), and absorption (e.g., “I am immersed in my work”). The UWES-9 consists of nine items rated on a 7-point scale from 0 (never) to 6 (every day). The three subscales are highly correlated, and the authors suggest that rather than computing three different scores, the total nine-item score of the UWES-9 can be used as an indicator of work engagement. Several studies using confirmative factor analysis have demonstrated the factorial validity of the UWES-9 (Richardsen et al., 2006; Schaufeli et al., 2006; Balducci et al., 2010). Vîrgǎ et al. (2009) conducted a translation process and validation study on Romanian population, sustaining good validity and fidelity for the UWES-9. The reliability analysis we performed on our sample revealed a Cronbach’s alpha coefficient of 0.817.

*Job crafting* (JC) was measured by using three dimensions of the Job Crafting scale developed by Tims et al. (2012). The following dimensions were used: (1) increasing structural job resources (e.g., “I try to learn new things at work”) including five items; (2) increasing social job resources (e.g., “I ask my supervisor to coach me”) with five items and (3) increasing challenging job demands (such as “When there is not much to do at work, I see it as a chance to start new projects”), comprising five items. The scale was rated on a 5-point Likert scale from 1 (never) to 5 (often). This measure was adapted and validated on Romanian population (Oprea and Ştefan, 2015), revealing a good internal consistency for all dimensions (alpha Cronbach between 0.65 and 0.79). The reliability analysis for our sample revealed a Cronbach’s alpha coefficient of 0.876.

*Performance* (P) was measured with the six items previously used by Mastenbroek et al. (2014) from the scale developed by Goodman and Syvantek (1999). It consists of two subscales: in-role performance, three items (e.g., “You meet all the requirements of your position”) and extra-role performance, three items (e.g., “You help your colleagues with your work when they return from a period of absence”). The responses are presented on a 7-point Lickert scale, from 0 (“not at all characteristic”) to 6 (“totally characteristic”). The scale was translated to Romanian language through the process of retroversion. For our sample, the statistic indicators show a good internal validity and support for the factorial structure of two factors. The reliability analysis we performed on our sample revealed a Cronbach’s alpha coefficient of 0.910.

### Statistical Analyses

Statistical analyses were performed using IBM SPSS AMOS 20.0.0 (IBM, 2011) and Jamovi 1.2.2.0 (The Jamovi Project, 2020). First, participants with missing values on one or more of the used scale items were removed from the analysis. In the next step outliers were removed thus reducing the size of the valid sample to *n* = 1151. In the next phase, the proposed research model was tested using structural equation modeling (SEM/maximum likelihood) with the AMOS software package. Latent variables job crafting, work meaning, work engagement, and performance were modeled with the corresponding scale factors (i.e., increasing structural job resources, increasing social job resources, increasing challenging job demands, positive meaning, meaning making through work, greater good motivations, vigor, dedication, absorption, in-role, and extra-role). In order to assess and test the specific indirect effects between variables we used the bootstrap analysis option in AMOS. The null hypothesis, that the predictor variable has no indirect effect on the outcome variable via the mediator variable, is rejected when the whole confidence interval computed by the bootstrapping method lies above or below zero. To assess the model fit we used the χ^2^-statistic, the comparative-fit-index (CFI), and the root mean square error of approximation (RMSEA) with 90% confidence intervals. For good model fit we considered values of CFI to be ≥0.95 and RMSEA to be ≤0.06 (Hu and Bentler, 1999).

## Results

### Descriptive Statistics

The descriptive statistics, including the means, standard deviations, and correlations of the study variables can be found in Table 1. All dimensions of the study variables were positively correlated to each other.

**TABLE 1:** Descriptive statistics and inter-correlations of the study variables.

	Mean	SD	1	2	3	4	5	6	7	8	9	10
1. Increasing structural job resources	18.70	3.33	−									
2. Increasing social job resources	12.84	3.62	0.413**	−								
3. Increasing challenging job demands	15.66	3.83	0.669**	0.521**	−							
4. Positive meaning	17.41	2.02	0.410**	0.199**	0.373**	−						
5. Meaning making through work	12.88	1.64	0.395**	0.219**	0.356**	0.882**	−					
6. Greater good motivations	12.93	1.80	0.296**	0.120**	0.255**	0.701**	0.619**	−				
7. Vigor	15.38	2.38	0.290**	0.185**	0.285**	0.378**	0.376**	0.210**	−			
8. Dedication	15.90	1.99	0.351**	0.180**	0.311**	0.454**	0.440**	0.285**	0.731**	−		
9. Absorption	14.21	2.53	0.206**	0.166**	0.272**	0.275**	0.268**	0.199**	0.476**	0.475**	−	
10. In role performance	15.11	2.79	0.517**	0.180**	0.393**	0.364**	0.318**	0.273**	0.346**	0.361**	0.187**	−
11. Extra role performance	15.04	2.77	0.499**	0.253**	0.430**	0.369**	0.332**	0.316**	0.307**	0.342**	0.257**	0.745**

*n = 1,151, ^∗^p < 0.05, ^∗∗^p < 0.01.*

### Structural Equation Modeling

**FIGURE 2:**
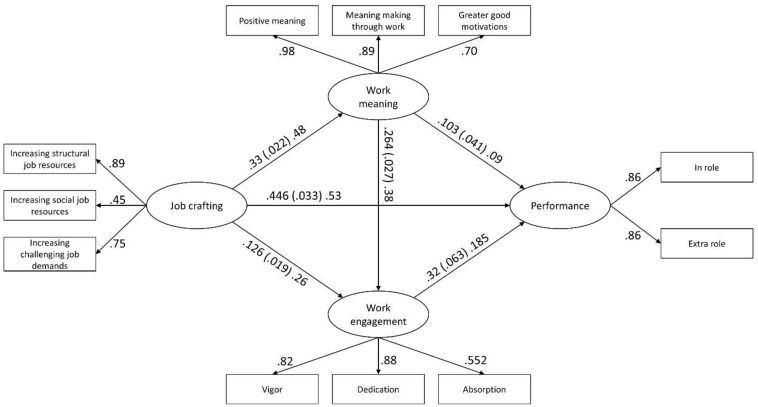
Results of the proposed research model. Estimates are reported as “unstandardized (standard error) standardized” and “standardized.” All reported coefficients and factor loadings were significant (<0.001).

We tested the full model, as presented in Figure 1, using the AMOS software package. In order to improve the model fit we allowed for the covariance of residuals for the increasing social job resources and increasing challenging job demands factors. The tested model showed a good fit with the data in relation to our proposed index references [χ^2^ (37) = 141.534, *CFI* = 0.985, *RMSEA* = 0.0.5, *CI* (0.041, 0.059)]. The estimates of our proposed research model are illustrated in Figure 2. The results were in accordance with the hypothesized relationships. Results showed that job crafting was positively related to work meaning (h1) (β = 0.48, ρ < 0.001), to work engagement (h2) (β = 0.26, ρ < 0.001) and to performance (h5) (β = 0.53, ρ < 0.001). Furthermore, work meaning was significantly related to work engagement (h3) (β = 0.38, ρ < 0.001). Finally, there was also a direct effect from work engagement to performance (h6) (β = 0.18, ρ < 0.001). These findings offer support for our hypothesized relationships.

In order to examine the indirect effects proposed in our hypotheses we used the bootstrap analysis option in the AMOS software package. Relating to h1, we found that job crafting had a stronger effect on the positive meaning [estimate = 0.47, ρ = 0.006, B-CCI (0.43, 0.52)] and meaning making through work [estimate = 0.425, ρ = 0.009, B-CCI (0.382, 0.462)] factors than on the greater good motivations factor [estimate = 0.337, ρ = 0.005, B-CCI (0.301, 0.383)]. Specifically, we tested three indirect effects. First, we tested the indirect effect of job crafting on work engagement via work meaning (h4). The results of the bootstrap analysis showed that this indirect effect was significant [estimate = 0.180, ρ = 0.006, *B-CCI* (0.159, 0.216)], thus offering evidence for hypothesis 4. Second, we tested the indirect effect of work meaning on performance through work engagement. This indirect effect was also statistically significant [estimate = 0.07, ρ = 0.007, *B-CCI* (0.041, 0.099)]. The direct effect of work meaning on performance, similar to the indirect effect, was weak (β = 0.09, ρ < 0.05). These results partially confirm hypothesis 8 but both effects are very weak.

Overall, the indirect effect of job crafting on performance was significant [estimate = 0.122, ρ = 0.006, *B-CCI* (0.092, 0.157)]. Taken together, these results offer support for a partial mediation effect from job crafting to performance through work meaning and work engagement. In total, the model predicted 47% of the variance in performance.

Next, we tested two alternative models derived from our proposed research model. The first model included only the direct relationships of job crafting, work meaning and work engagement with job performance. This alternative model showed a poor fit with the data [χ^2^ (40) = 689.406, *CFI* = 0.904, *RMSEA* = 0.120, *CI* (0.112, 0.128)]. The second model was focused on the indirect effects, so we excluded the direct path from job crafting to performance from the initial model. The results showed that the indirect effects model also presented a poor fit with the data [χ^2^ (38) = 352.288, *CFI* = 0.954, *RMSEA* = 0.085, *CI* (0.077, 0.094)]. In conclusion, these results offer additional support for the proposed research model.

### Additional Analyses

Even if, so far, we found support for our proposed model, the question remains whether the different dimensions of job crafting have different contributions within the model. In order to analyze this issue, we tested three additional models in which the latent job crafting variable was replaced with a manifest variable as follows: (M1) increasing structural job resources, (M2) increasing social job resources, (M3) increasing challenging job demands. All models showed good fit with the data. The comparative fit results of the analyses can be seen in Table 2.

**TABLE 2:** Goodness of fit indices of additional models (*n* = 1,151).

Model	RMSEA	χ^2^	*df*	CFI
M1	0.049	82.501	22	0.989
M2	0.056	99.365	22	0.985
M3	0.053	91.556	22	0.987

*χ^2^ = chi-square; df = degrees of freedom; RMSEA = root mean square error of approximation; GFI = goodness-of-fit index.*

For the first model we observed a small increase in the indirect effect from increasing structural job resources to performance [estimate = 0.14, ρ = 0.005, B-CCI (0.117, 0.174)]. Increasing structural job resources was positively related to work meaning (β = 0.42, ρ < 0.001), to work engagement (β = 0.21, ρ < 0.001), and to performance (β = 0.45, ρ < 0.001). This model accounted for 42% of the variation in performance. In the second analysis results indicated a smaller indirect effect from increasing social job resources to performance compared to the first model [estimate = 0.117, ρ = 0.011, B-CCI (0.092, 0.143)]. Increasing social job resources was positively related to work meaning (β = 0.20, ρ < 0.001), to work engagement (β = 0.12, ρ < 0.001) and to performance (β = 0.14, ρ < 0.001). This model accounted for 28% of the variation in performance. The indirect effect from increasing challenging job demands to performance within the last model was the largest of all three tested models [estimate = 0.16, ρ = 0.008, B-CCI (0.137, 0.192)]. Increasing challenging job demands was positively related to work meaning (β = 0.38, ρ < 0.001), to work engagement (β = 0.20, ρ < 0.001), and to performance (β = 0.32, ρ < 0.001). This model accounted for 35% of the variation in performance. These findings suggest that increasing structural job resources was the most important factor in our proposed model.

## Discussion

The goal of the current study was to expand the knowledge regarding the motivational process of firefighters by employing job crafting and introducing the concept of work meaning within the motivational framework of the JD-R model. In this article we argued that, given the dangerous nature of the work of firefighters and their strong sense of group identity, job crafting would play an important role in sustaining engagement and work meaningfulness. We hypothesized that individuals who craft their jobs are more likely to experience higher levels of meaningfulness and engagement in their work, which subsequently leads to increased performance. Job crafting had a positive relationship with perceived job performance, both directly and indirectly, through work meaning and work engagement, the latter effect being substantially weaker than the former. Overall, the model explained 47% of the variance in job performance.

### Contributions

A first contribution of the current study is that we found evidence for the existence of job crafting behaviors among firefighters. More so, our results revealed that job crafting is an important aspect within firefighters as it was directly responsible for increases in job performance. Specifically, increasing structural job resources was the highest contributor to job performance in our proposed model. This result is in accordance with previous research (Bakker and Demerouti, 2014; Rudolph et al., 2017) and may be due to the fact that the difficult job demands that firefighters face stimulate the process of resource seeking which in turn facilitates performance. Further research is needed in order to better differentiate the effects that different dimensions of job crafting have on job performance within firefighters by employing a more specific model relating to job crafting behaviors and performance outcomes among firefighters.

A second contribution of this study is that it shows a relationship between job crafting and work meaning. This result is consistent with research emphasizing the fact that work meaning is not only a consequence of job characteristics but also a result of individual (re)designing of the tasks and relational boundaries in their job (Leana et al., 2009; Rosso et al., 2010). Related to this is the fact that, in our study, job crafting had a larger effect on the perceived meaningfulness of work (positive meaning and meaning making through work), rather than on the actual meaning that the work of firefighters has (greater good motivations). This could be explained by the fact that the social importance of firefighters’ work (greater good motivations) is mostly a result of job characteristics and as such, it could be less prone to individual shaping.

Another important result relates to the positive relationship between work meaning and work engagement. This result is in accordance with findings in previous studies (Van Zyl et al., 2010; Geldenhuys et al., 2014) in which meaningful work predicted more engaged employees. According to Britt et al. (2007) one of the reasons why individuals engage in certain work activities is because they experience a meaningful relation to central aspects of their self-concept. Additionally, given that firefighters constitute a specific group with a particular identity and that their activity is of great social significance and implies teamwork, the relation between work meaning and work engagement could be explained through a social identity theory lens (Tajfel and Turner, 1985). The psychological identification with the group and the arching work community becomes an important source of meaning (Katz and Kahn, 1978; Weick, 1995) and also provides an important incentive to engage in activities and to perform in a manner that will ensure the preservation and strengthening of the affiliation with the group.

Related to these findings, we found support for an alternative mean by which job crafting can impact work engagement, that is, through work meaning. This indirect effect was significant and similar in size to the direct effect from job crafting to work engagement. By engaging in job crafting behaviors firefighter personnel may obtain a greater sense of meaningfulness and in doing so they are able to maintain a necessary level of engagement that is critical in the exercise of their work. Also, by crafting their work individuals can gain access to more resources or develop new challenges that stimulate and focus their personal resources and engagement.

Unexpectedly, in our sample, results revealed a weak predictive power for work engagement in regard to job performance. Further research could shed more light on this result and better determine its origins and significance. This aspect consequently led to weak overall indirect effects on performance suggesting possible additional mechanisms by which job crafting and work meaning influence job performance. As such, work meaning and its significance in relation to performance outcomes needs further clarification among samples of firefighters. Future studies could be directed toward a better understanding of the process by which work meaning relates to the interaction between the organization and the individual and to determine its impact on the organizational and personal outcomes. Nevertheless, our results suggest that firefighters are engaged in job crafting, thus shaping their work meaning and improving work engagement and performance.

### Limitations

The methodological issues concern firstly the cross-sectional approach we used in this study. A number of potential problematic aspects arise from this approach. One such issue might be that our design only allowed for associations between the measured variables and not for assumed causality. Such claims could be discussed in the case of a longitudinal study or in experimental approaches to the proposed theme. A longitudinal research design should offer the opportunity for better insight on causal attributions concerning the role of work meaning within firefighters. Also, the use of designed interventions that pertain to job crafting might be useful in determining changes in work meaning and organizational outcomes.

Another issue concerns the potential bias in our data given that we used self-reported measures. The data originated from the same person at a given moment, so the relationship between the measured variables could have been artificially impacted due to certain aspects that are characteristic of responders, such as need for consistency in responses, affective mood, socially desirable responding or lack of involvement that can lead to inaccurate or contradictory responses (Podsakoff et al., 2003). Further, we had no additional way of testing for the accuracy of the reported results. This issue could be improved in future studies, for example, by using colleague-ratings of observable variables within the selected sample and/or by requesting factual data about individual work results and activities that relate to the theme in question.

A third limitation of this study is the use of only one instrument for measuring work meaning. Given the lack of consensus in the research field on how this concept is currently defined and measured, a better approach would have consisted in the adoption of several instruments in order to cover the multiple aspects of this construct.

### Implications

This study raises the attention on ways in which Fire departments may be able to create a climate that emphasizes meaningfulness and engagement, together with job crafting opportunities. In line with this idea, Evans and Steptoe-Warren(eds) (2019) argue that first responders’ jobs are most likely to be in the future more open toward job redesign, a focus process based on efficiency gain. For instance, by describing the role their work has within a larger context, organizations could help workers in crafting work meaning (Vuori et al., 2012). The organizational climate would also need to be responsive to their needs, in a way that could make them ready to invest their resources in work and become more engaged. By crafting their work, firefighters are attributing meaning and they are shaping interpersonal contacts, contributing to team spirit. Team work is especially important in firefighters. The good functioning of the team can lead to better performance, given that firefighter’s missions imply a close reliance on each other in order to fulfill the assigned tasks. Another role of the firefighters’ team is that it creates a sense of belonging, a sense of meaning and a safe place for sharing thoughts and experiences. These aspects are important, since the literature emphasizes the positive outcomes of work meaning, such as: greater engagement, the awareness of the importance, and value of the job, but also fewer turnover intentions and greater commitment to the organization (Steger et al., 2012). In terms of psychological support and psychological assessment of firefighters, unit, psychologists could take into consideration the aspects related to job crafting, work meaning and work engagement, since these are key factors within the motivational process. As a synthesis of practical implications mentioned above, we can identify a few directions, based on our findings: improving periodical psychological assessment and training policies; developing organizational strategies aiming at improving well-being and performance and facilitating change; fostering communication within teams and leadership support. Fire departments have resources, means and opportunities available to maintain the well-being and commitment of their employees.

## Conclusion

In conclusion, we replicated some of the previous findings concerning job crafting, work engagement, and performance and we also introduced work meaning as an additional factor in explaining the relationships between these constructs. Interestingly, in our model we found evidence for the assumption that job crafting plays a substantial role in performance outcomes, even if firefighters’ jobs are presumably not prone to job crafting in its classical top-down approach (Oldham and Fried, 2016). Also, we found support for the idea that work meaning can be an outcome of job crafting and that greater levels of work meaning were associated with increased work engagement, and performance. Our findings encourage future research on how work meaning can add to more engaged, resilient and committed employees, on how organizations can foster meaning through characteristics compatible with employee job crafting and also on the role of job crafting and work meaning within the health-impairment process of the JD-R model.

## Data Availability Statement

The datasets generated for this study will not be made publicly available due to institutional constraints. Requests to access the datasets should be directed at cristinaioanadan@yahoo.com.

## Ethics Statement

Ethical review and approval was not required for the study on human participants in accordance with the local legislation and institutional requirements. Written inform consent of participation (concerning the aim of the study, the anonymity of data, and the use of data) was obtained prior the completion of the questionnaires.

## Author Contributions

All authors have made substantial and equal contribution in all stages of the present study. All authors read and approved the final version of this article.

## Conflict of Interest

The authors declare that the research was conducted in the absence of any commercial or financial relationships that could be construed as a potential conflict of interest.
